# MDLSD: study protocol for a randomised, double-masked, placebo-controlled trial of repeated microdoses of LSD in healthy volunteers

**DOI:** 10.1186/s13063-021-05243-3

**Published:** 2021-04-23

**Authors:** Robin J. Murphy, Rachael L. Sumner, William Evans, David Menkes, Ingo Lambrecht, Rhys Ponton, Frederick Sundram, Nicholas Hoeh, Sanya Ram, Lisa Reynolds, Suresh Muthukumaraswamy

**Affiliations:** 1 School of Pharmacy, Faculty of Medical and Health Sciences, University of Auckland, 85 Park Road, Grafton, Auckland, 1023 New Zealand; 2 Mana Health, 7 Ruskin St, Parnell, Auckland, 1052 New Zealand; 3 Department of Psychological Medicine, Faculty of Medical and Health Sciences, Waikato Clinical Campus, Peter Rothwell Academic Centre, University of Auckland, Pembroke Street, Hamilton, 3240 New Zealand; 4 Regional Cancer & Blood Service, Auckland District Health Board, 2 Park Road, Grafton, Auckland, 1023 New Zealand; 5 Department of Psychological Medicine, Faculty of Medical and Health Sciences, University of Auckland, 2 Park Road, Grafton, Auckland, 1023 New Zealand; 6 Department of Psychological Medicine, Faculty of Medical and Health Sciences, University of Auckland, 22-30 Park Avenue, Grafton, Auckland, 1023 New Zealand

**Keywords:** Microdosing, Lysergic acid diethylamide, Psychedelics, Cortical plasticity, Cortical connectivity, Personality, Creativity, Long-term potentiation, Randomised controlled trial

## Abstract

**Background:**

Regular ingestion of sub-hallucinogenic doses of psychedelics, referred to as “microdosing”, has gained increasing popularity and attention in the press and in online forums, with reported benefits across multiple cognitive and emotional domains. Rigorously controlled studies to date, however, have been limited in scope and have failed to produce results comparable to those reported in the grey literature.

**Methods:**

Eighty healthy male participants will receive 14 doses of placebo or 10 μg lysergic acid diethylamide orally every 3rd day over a 6-week treatment protocol. A battery of personality, creativity, mood, cognition, and EEG plasticity measures, as well as resting-state fMRI imaging, will be administered at baseline and at the end of the protocol. Creativity, mood, and plasticity measures will additionally be assessed in the acute phase of the first dose. Daily functioning will be monitored with questionnaires and a wearable sleep and activity tracker.

**Discussion:**

This study will rigorously examine the claims presented in the microdosing grey literature by pairing a comparable dosing protocol with objective measures. Potential therapeutic implications include future clinical trials to investigate microdosed psychedelics as a standalone treatment or as an augmentation of psychotherapy in the treatment of depression, addiction, eating disorders, obsessive-compulsive disorders, and palliative care.

**Trial registration:**

ACTRN12621000436875. Registered on 19 February 2021

**Supplementary Information:**

The online version contains supplementary material available at 10.1186/s13063-021-05243-3.

## Background and rationale

“Microdosing” refers to repeated administration of psychedelics such as lysergic acid diethylamide (LSD) or psilocybin in doses below the threshold for overtly altering perception [1]. A growing online microdosing subculture and resultant media interest claims wide-ranging benefits to mood, focus, creativity, self-efficacy, energy, sociability, cognition, psychological, and physiological well-being, with limited side effects [2]. These claimed benefits are similar to those observed in full dose clinical studies, in which participants receiving much larger, perception-altering, doses of LSD or psilocybin have demonstrated changes to mood [3], personality [4], and general feelings of connectedness and acceptance [5]. Despite a growing grey literature, preliminary randomised controlled trials have captured some physiological, psychological, and cognitive effects, but have largely failed to detect some of the broader claims [6–12], suggesting that they may in part be due to the influence of placebo and expectancy effects on self-reported data [13]. However, the trials conducted to date have been limited to single or a low number of repeated doses administered in laboratory settings, lacking the ecological validity of long-term home dosing. It is possible that these lab-based protocols lack the duration and environmental stimulation necessary to see measurable effects. The present study aims to address this gap in the literature by administering fourteen 10 μg doses of LSD (or inactive placebo) over a 6-week protocol, with all but the first dose self-administered by participants in their own homes.

### Microdosing practises

First popularised by the publication of an every 3-day dosing protocol in James Fadiman’s 2011 book *The Psychedelic Explorer’s Guide* [14], microdosing has received attention in the mainstream press [15] as well as in online forums [16] and on YouTube [17]. The prevalence of microdosing practises in the general population is not known; however, an online recreational drug use survey found that 13% of respondents had microdosed at some point and that 4% were currently microdosing [18]. Content analysis of online self-reports and surveys of existing microdosers have identified that motivations and claimed benefits of microdosing fall into the broad categories of self-optimisation/improvement, self-treatment, and, to a lesser extent, self-exploration [2, 17, 19]. As well as purportedly boosting productive capabilities such as focus, creativity, and athletic performance; microdosers promoting the practice on YouTube have reported greater presence at the moment, with the drug serving as a “non-specific amplifier” of experience [17]. Self-treatment benefits were attributed by microdosers to this effect, in that enhanced presence facilitates access to self-insight and allows microdosers to work through existing issues instead of masking them, as they believe other psychiatric medications do [17]. This is similar to beliefs identified in a clinical trial of treatment-resistant depression with full dose psilocybin therapy. Patients felt that psychedelic therapy increased their feelings of connection and acceptance in contrast to other therapies which enhanced disconnection and avoidance [5]. However, there is a substantial risk of bias among these self-reports. Expectancy of positive benefits has been shown to be high among those who participate in online microdosing forums [20]. These positive beliefs about the practice have been shown to correlate with self-reported improvements in mood and well-being among community microdosers [13]. A recent study, which used a novel self-blinding protocol with participants sourcing their own drug materials, found that correcting for the number of times participants guessed they had taken an active microdose reduced already insignificant differences between the placebo and drug groups in self-reported measures, but not more objective cognitive measures, suggesting that self-reports are indeed likely susceptible to placebo effects [21]. These results necessitate placebo-controlled investigations which account for expectancy and beliefs around microdosing at baseline.

### Mechanism of action

LSD is a serotonergic psychedelic, which in large doses causes significant perceptual changes and an altered state of consciousness. The primary mechanism of action of serotonergic psychedelics is thought to be by its partial agonism of the serotonin 2A (5HT_2A_) receptor [22] as the subjective effects of LSD are blocked by the relatively selective 5HT_2A_ antagonist ketanserin [23]. It has been theorised that the subjective effects of LSD are instigated by potentiation of 5HT_2A_ receptor-dense layer V pyramidal cells, leading to a disintegration of typical functional network connectivity [24]. Imaging studies of full doses of serotonergic psychedelics show widespread connectivity changes, in particular, a disintegration of the default mode network (DMN) [25]. Decreased connectivity between the parahippocampal gyrus and restrosplenial cortex within this network has been shown to correlate with subjective ratings of “ego dissolution” and “altered meaning” [25], suggesting that changes to DMN connectivity drive the consciousness-altering effects of psychedelics. To date, there has been only one imaging study on the effects of microdosing, restricted to the acute phase of the psychedelic dose. This study found changes in connectivity to the thalamus and amygdala, with increases in connectivity between the amygdala and the right middle frontal gyrus positively correlating with increases in positive mood following 13 μg of LSD, a quantity around one tenth of that needed to create a full dose psychedelic “trip” [6]. While consciousness-altering effects such as ego dissolution are not expected to occur under microdosing, functional imaging following repeated microdosing could provide insight into whether other benefits of the practice are due to network-level functional alterations.

### Personality

Prior to the legal scheduling of psychedelic drugs in the USA in 1966, research indicated that the administration of large doses can cause enduring changes in outlook and personality [14], claims that have been examined more recently using measures of the “big five” personality traits. In particular, the trait of openness has repeatedly been shown to increase following psychedelic doses, while changes to the traits of neuroticism, extraversion, and conscientiousness vary inconsistently [4, 26, 27]. One study found that in those participants who had a mystical experience, the increase in openness was sustained for longer than 1 year [4]. While the effects of long-term microdosing on personality have not yet been studied in a controlled environment, one observational study, which tracked community microdosers over 6 weeks, found a significant increase in not only openness, but also neuroticism [20]. This study also found an increase in the trait absorption, as measured by the Tellegen Absorption Scale (TAS) [28]. Absorption refers to a tendency for one’s attention to become deeply absorbed in sensory experiences and has been shown to predict the phenomenological intensity of the effects of full dose psychedelic “trips” [29]. These personality changes are consistent with anecdotal reports collected by Fadiman [14], which suggest that there is a “gradual build-up of openness and awareness” with microdosing (p211). However, a similar recent study [13] found no significant change to openness or absorption after 4 weeks of microdosing but did find increases in emotional stability, a construct not explicitly measured in the prior study. Both of these studies are limited by uncertainty around dosage as they relied on participants sourcing and preparing their own drug materials.

### Creativity

Mid-twentieth century studies also examined creativity under full doses of psychedelics, reporting that participants were able to find novel and useful solutions to technical problems [30] and showed freer artistic expression [31]. It has been suggested that psychedelics put participants into hyper-associative states, free of the logical constraints which usually limit creative thinking, potentially driven by alterations in network connectivity [32]. While creativity is a notoriously contentious value to measure [33], attempts to objectively verify the self-reported effects of microdosing on creativity have detected an effect. A naturalistic study of the effects of low-dose psilocybin truffles demonstrated increases in both convergent and divergent thinking during the acute phase of the dose [34]. Convergent thinking represents the ability for associative spread to reach a pre-determined answer, and divergent thinking represents the ability to think of novel answers [35]. In a similar task undertaken via survey, current and former microdosers scored higher on measures of creativity than controls [36]. Together these results suggest that even small doses of psychedelics may allow people to access similar unconstrained, hyper-associative states to those experienced under full doses.

### Cognition

Standardised cognitive tasks during the acute phase of microdosing have largely failed to detect effects of the magnitude reported in the grey literature. The Dual N-Back (a measure of fluid intelligence) and Digit Symbol Substitution Task (DSST; a measure of attention, motor speed, working memory and visual processing) failed to show any effect following doses of 6.5, 13, and 26 μg of LSD [7], neither did the Raven’s Progressive Matrices Task (a measure of fluid intelligence) following psilocybin truffles [34] nor the Cambridge Neuropsychological Test Automated Battery (a broad battery of cognitive function tests) after 5, 10, and 20 μg LSD [8]. However, participants who underwent the Psychomotor Vigilance Task (a measure of reaction time and attention) did show fewer attentional lapses after taking 5 and 20 μg doses (but not 10 μg doses), suggesting that increased attention and focus reported by community microdosers may plausibly be detected in laboratory settings [37]. There may also be slight cognitive deficits associated with the acute phase of higher microdoses, as the same study found that participants made significantly fewer correct responses on the DSST after 20 μg doses. However, this effect was no longer significant after correcting for the number of responses overall, suggesting that it was due to reduced responses, rather than reduced accuracy. Both increased attention and reduced response rates may be the result of altered time perception, as an overestimation of temporal intervals in the 2000–4000 ms range has been detected following 10 μg doses of LSD [9]. This effect occurred independent of subjective ratings of the drug’s effects and as such could reflect neurobiological processes that occur even at sub-perceptual doses and therefore independent of a placebo effect. It is plausible that broader cognitive changes reported in the grey literature will not be measurable until after repeated microdoses in an ecologically valid setting.

### Mental and physical well-being

Retrospective surveys of people who have microdosed consistently cite mental health improvements as a motivation for and outcome of microdosing [2, 13, 18, 20, 38, 39]. One survey of people who have microdosed found that 39% were motivated by self-treatment of pathologies including depression, anxiety, attention deficit hyperactivity disorder (ADHD), post-traumatic stress disorder (PTSD), and substance dependence [39]. Among these respondents, nearly 90% rated the practice as helpful and only 1.7% rated as unhelpful, as opposed to antidepressants, which only 35.5% rated as helpful and 53.9% unhelpful. Non-clinical reports of improved mood are consistent across microdosing survey reports [2, 38–40]. Tracking a community of people microdosing using validated subjective measures has shown significant increases in mental well-being and decreases in depression and anxiety over 4 weeks of microdosing [13] and significant decreases in depression and stress symptoms over 6 weeks of microdosing [20]. Daily tracking of participants in the 6-week study showed they rated themselves as significantly happier on dose days, falling back to near baseline levels in the subsequent 2 days after dosing [20].

Microdosing has also been used in the community for chronic physiological conditions such as migraines, cluster headaches, and chronic pain, with participants significantly more likely to rate this practice as effective when compared to conventional treatments [40]. While no controlled studies of these effects have been undertaken with clinical populations, a recent study found that following doses of 20 μg LSD, participants were able to tolerate a cold water pain test longer, accompanied by lower ratings of pain and discomfort, suggesting that there may be an analgesic or pain tolerance effect present even at fairly low doses [12].

### Plasticity

While some people who have microdosed report effects that persist in the days following microdoses [14], the half-life of microdoses of LSD is only ~ 8 h [8] suggesting that long-term effects may be caused by downstream mechanisms triggered by the dose. These cognitive changes may be driven by changes to structural plasticity. Cortical neurons in vitro have shown increases in structural plasticity following exposure to serotonergic psychedelics including increased complexity of dendritic arbours (neuritogenesis), spine growth (spinogenesis), and synapse formation (synaptogenesis) [41]. Additionally, the serotonergic psychedelic 2,5-dimethoxy-4-iodoamphetamine (DOI) has been observed to induce neurogenesis in the hippocampus [42]. In the neocortex, administration of DOI results in upregulation of the expression of both *Arc* mRNA and brain-derived neurotrophic factor (BDNF) mRNA [43]—both of which code for proteins that are important for experience-dependent plasticity. BDNF in particular has a well-established role in structural neuroplasticity mechanisms such as synaptogenesis [44] and a demonstrated relationship with functional plasticity and long-term memory [45]. While the directly measurable plasticity changes described above were observed following a full psychedelic dose administered to rats or rat neurons, a recent human in vivo study found that LSD doses as low as 5 μg significantly increased circulating plasma levels of BDNF at least 6 h after dosing [11], suggesting that neurotrophic changes following microdoses could plausibly persist beyond the drug’s presence in the body; however, research extending the window of observation is needed to confirm this.

While cellular mechanisms of plasticity cannot be directly measured in humans non-invasively, electroencephalography (EEG) recording, combined with sensory processing tasks, has been used to indirectly assess the state of plasticity in the human brain [46]. Two of the most commonly measured forms of plasticity include long-term Hebbian plasticity and shorter-term predictive coding [46]. Hebbian plasticity—an increase in synaptic connectivity in response to repeated stimulation—can be indexed by visually inducing long-term potentiation (LTP) and measuring the consequent modulation of visually evoked potentials [47]. Secondly, predictive coding—identification and adaptation to novel or unexpected input—can be indexed via the auditory Roving Mismatched Negativity (MMN) paradigm [48] and the consequent modulation of auditory evoked potentials. Each offers important and unique information on the state of plasticity in the human brain [46]. These tests have not yet been administered following repeated home microdosing.

### Safety and tolerability

Unwanted dose day experiences reported in a survey of people who have microdosed include difficulty concentrating, feeling overwhelmed, overstimulation, difficulty sleeping, and euphoria and the feeling of “tripping” [38]. Many of those surveyed reported experiencing these effects at least once, but few reported them occurring after every dose. Other reports note that negative effects are largely acute and rarely persist in the long term [19]. Despite these effects, when people who had microdosed were asked why they had quit the practice, the most cited reasons were practical, in particular, the risks and challenges of obtaining an illegal substance [18].

Laboratory studies have shown that the subjective experience of microdosing accurately measured amounts of pharmaceutical-grade LSD is minimal. In one study, the 5-Dimensional Altered States of Consciousness Questionnaire (5D-ASC), a widely used measure of perception and consciousness-altering effects, did not show significant changes following 13 μg doses of LSD, and more participants guessed that they had received a placebo or a sedative than a psychedelic; however, participants did show a small but significant increase to systolic blood pressure (BP), which remained within a healthy range, with no change to diastolic BP or heart rate [6]. In a different study, the 5D-ASC showed changes along multiple dimensions following 20 μg doses and changes along the dimension of anxious ego dissolution for 10 μg; however, a specific measure of ego dissolution showed no effect for either dose group [37]. There are no reports of serious adverse events (SAEs) resulting in hospitalisation or death in the literature. While older adults who received doses of 5, 10, and 20 μg of LSD reported a wide range of adverse events in the 8–12 h after dosing, the only effect that showed a significant difference between the placebo and treatment groups was reports of headache, of which all were mild or moderate [8].

### Explanation for the choice of comparators

The current microdosing protocol of 10 μg LSD every third day was intended to replicate the typical practices of microdosing in the community as closely as possible. Among the classical psychedelics, LSD and psilocybin are the most commonly used for microdosing, with one survey study finding psilocybin use slightly more common [38], but most showing a greater prevalence of LSD [2, 18, 19, 49]. Accurate measurement of psychedelic doses is difficult for people microdosing outside of laboratory environments. Estimated LSD microdoses range widely when estimated by these participants, but doses estimated at 10–13 μg are most commonly reported, representing ~ 10% of a full dose “trip” [19, 38, 49]. While many people optimise their own schedule [19], the dosing schedule of every third day outlined in Fadiman’s influential book [14] remains standard [16, 49].

While an active placebo, such as caffeine, may help to prevent unmasking (masking here is used in place of “blinding” to distinguish from studies of visual impairment), home-dosing poses safety challenges as participants will be given four or five doses at a time to store and administer at dosing points. In order to prevent the possibility of non-compliant participants taking a large dose of the active placebo if they attempted to achieve an LSD “high”, an inactive placebo was chosen instead. In order to mitigate the possibility of participants being unmasked by the perceptual effects of the microdose, a parallel-group design has been chosen instead of a crossover design. In order to prevent a nocebo effect if participants do not experience perceptual effects, participants will be informed that many people do not experience any noticeable effects from a microdose. Unmasking will be monitored by asking participants at each dose day to guess whether they have taken an active or placebo dose.

### Objectives

To examine the self-improvement benefits suggested in self-reports, we will assess the measures of personality structure and creativity. Specifically, open-mindedness and the related construct of absorption, as well as divergent and convergent thinking will be measured. Our hypotheses here are that participants will show increased open-mindedness compared to placebo as measured by the Big Five Inventory-2 (BFI-2) [50] and absorption as measured by the Modified Tellegen Absorption Scale (MODTAS) [28, 51], and increased divergent thinking as measured by the Alternate Uses Test (AUT) [35] and convergent thinking as measured by the Remote Associates Task (RAT) [52].

To assess the possible neural mechanisms of these changes, we will use established measures of cortical plasticity and connectivity. We hypothesise that participants who receive LSD will show greater levels of plasticity than placebo, as measured by the LTP and MMN paradigms described by Sumner et al. [53, 54], and will show modification to the connectivity of the DMN as measured by analysis of within- and between-network correlations of node activity during resting-state fMRI.

Because of the early stage of the field, a comprehensive battery of secondary measures will be administered, including mood, cognition, mindfulness, flexibility, peripheral blood mononuclear cell (PBMC) biomarkers, inflammatory cytokines, drug plasma levels, and supplementary creativity, personality, and connectivity measures (see Table 3). Analysis of these secondary measures will be considered exploratory, and reporting of any significant results will reflect the caution necessary in interpreting them appropriately.

## Methods/design

### Participants

All participants will be healthy males aged 25–60, with no recent history of psychedelic use. Participants will be screened according to the full inclusion and exclusion lists in Tables 1 and 2.

**Table 1:** Full inclusion criteria

	Inclusion criteria
Consent	Willing and able to give informed consent for participation in the trial, reconfirmed verbally at each study visit.
Demographics	
Age	25–60 years
Sex	Male

**Table 2:** Full exclusion criteria

	Exclusion criteria
Consent/communication	Inability to speak or read English
Physiological health	
Diagnosis	Unstable medical or neurologic condition as assessed by the study physician
Lab work	Significant renal or hepatic impairment
Vital signs	Cardiovascular conditions including abnormal heart rate seen by ECG Resting blood pressure not exceeding 160 mmHg systolic and 90 mmHg diastolic Body weight between 50 and 120 kg
Medical history	Contraindications for MRI scanning
Mental health	
Diagnosis	Lifetime history of major depressive disorder, schizophrenia or other psychotic disorders, or bipolar I or II disorder as assessed by the Mini International Neuropsychiatric Interview (MINI) Current diagnosis of PTSD, anxiety and panic disorders, OCD, dysthymic disorder, anorexia, and bulimia as assessed by the Standard MINI
Current risk	Elevated of suicide as determined by study psychiatrist using the Columbia-Suicide Severity Rating Scale (C-SSRS) Elevated risk of developing psychosis as determined by the study psychiatrist using the Comprehensive Assessment of At Risk Mental States (CAARMS)
Family diagnosis	First-degree relatives diagnosed with schizophrenia or other primary psychotic disorder, or bipolar I or II disorder
Medication	Current use of any prescribed psychotropic medication
Substance use	Substance use disorder in the previous 3 months as assessed with a New Zealand modified version of the NM-ASSIST Failed breathalyser and/or multipanel drug urine tests at screening with one follow up in trial Use of serotonergic psychedelic drugs in the last year Lifetime history of psychedelic microdosing

### Study design

This study is a randomised, participant and investigator-masked, inactive placebo-controlled parallel-group trial with 80 participants. Participants will be allocated into parallel groups in blocks of ten in a 1:1 ratio. Given the early stage of this field, an exploratory framework has been chosen. The study drug or placebo will be self-administered by participants from 1-ml oral syringes containing 10 μg of LSD or placebo (see the “Drug preparation and administration” section). Visits will take place at research facilities in the Faculty of Medical and Health Sciences on the Grafton Campus of Auckland University in New Zealand.

At a screening visit, volunteers will give informed consent, be checked for eligibility, and will be approved for inclusion by a study psychiatrist. Written informed consent will be obtained by members of the study team from the participants through the process outlined below. A participant information sheet (PIS) and informed consent form (see Additional File 1) will be supplied to prospective participants prior to their attendance at the screening visit, with adequate time to seek independent advice, for example, from a lawyer, general practitioner (GP), and relevant family members. These forms contain information on the nature of the trial, what involvement will entail for the participant, the implications and constraints of the protocol, the known side effects, and any risks involved in taking part. Participants will have the opportunity to ask questions of the study investigators prior to and again during the screening visit, and their verbal understanding of the information will be confirmed prior to giving written informed consent. Continuing eligibility and verbal consent will be reconfirmed at every study visit.

**Fig. 1:**
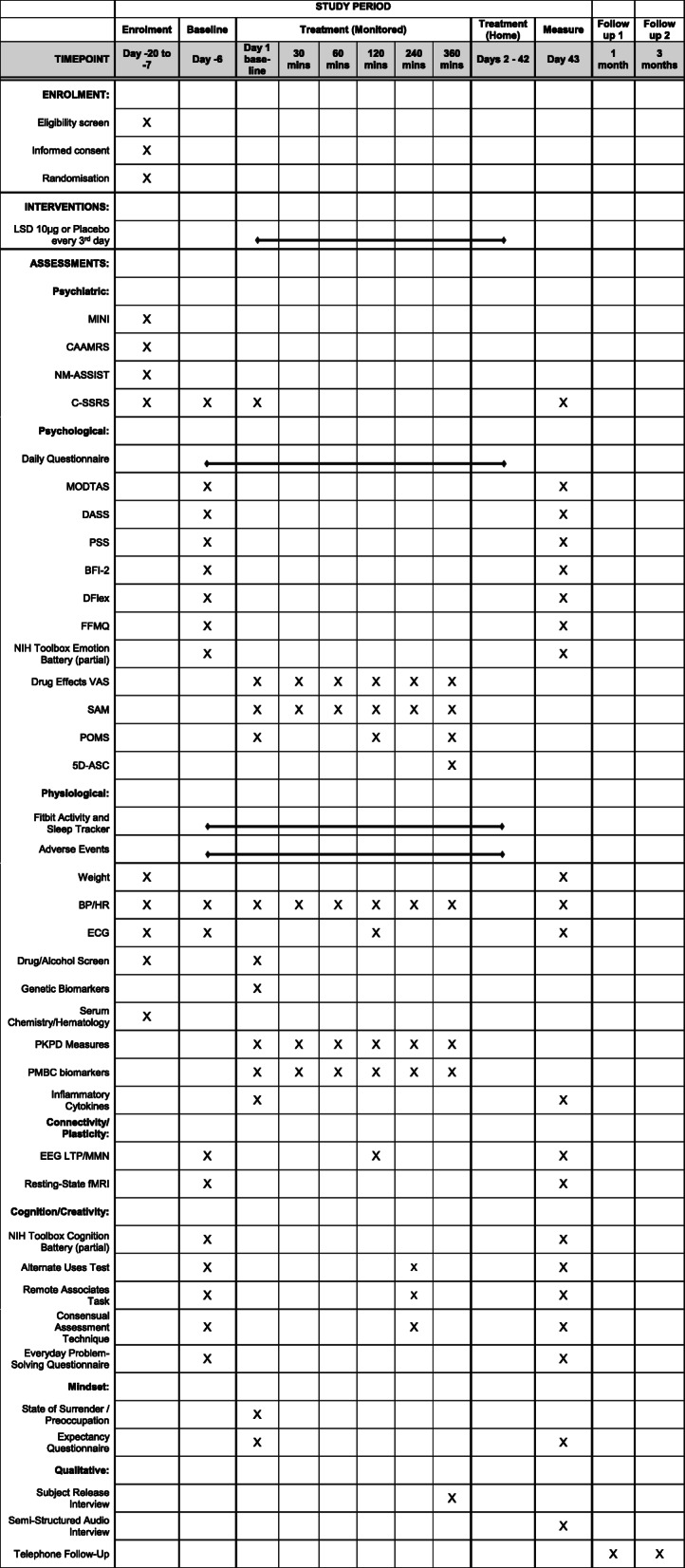
Schedule of enrolment, interventions, and assessments

Following acceptance to the trial, participants will return for a second visit to collect baseline measures (day − 6; see Fig. 1). The following evening, participants will receive a text message with a link to complete a questionnaire of visual analogue scale (VAS) ratings every day until the final study visit (day 43). One week later (day 1), participants will return to the lab to receive a single dose of their first allocated intervention and be monitored for 6 h before being discharged. Blood will be drawn prior to drug administration and at 30, 60, 120, 240, and 360 min after administration. Subjective drug effect measures will also be collected at these time points. EEG measures will be taken at ~ 150 min after administration and creativity measures at ~ 260 min. Participants will be discharged with four additional doses and will then self-administer oral syringes sublingually every third morning on 12 occasions and fourth morning on one occasion (days 4, 7, 10, 13, 16, 19, 22, 25, 28, 31, 34, 37, 41). Participants will make a brief re-supply/health check visit on days 14 and 26 and will receive 4 and 5 additional doses, respectively, on these dates. On day 43, all baseline measures will be repeated, as well as a qualitative interview. Brief follow-up telephone interviews will be conducted at 1 and 3 months.

Participants will be withdrawn from the trial and the intervention will be immediately ceased if the participant requests it, one of the exclusion criteria above is identified or violated, there is not adequate dose compliance, they experience a serious adverse event, they experience a persistent non-serious adverse event which interferes with their daily functioning, and any other condition emerges which is judged by the study team as likely to impact on the ability of the participant to complete the trial. Decisions about withdrawing participants will be made with the advice of study clinicians.

### Outcomes

The timeline of assessments is outlined in Fig. 1, with summaries of primary measures and secondary measures in Table 3. Questionnaires, including the primary personality measures (BFI-2 and MODTAS), will be self-administered (see the “Data collection and management” section) at the baseline and after the treatment period on day 43. BFI-2 measures five personality dimensions; however, only open-mindedness will be treated as a primary outcome, with the others being assessed as secondary outcomes. EEG and creativity measures will be repeated at baseline, in the acute phase of the first dose on day 1, and on day 43. fMRI will be repeated at baseline and day 43 only.

**Table 3:** Primary and secondary measures

Measure	Domain	Scale
Primary measures		
Alternate Uses Test (AUT) [35]	Creativity: divergent thinking	3 items; participants are given the name of a household item and are given 2 min to produce possible uses; responses are marked for fluency (number of responses), flexibility (number of different categories of responses), elaboration (number of elaborative details), and originality (uniqueness of response).
Big Five Inventory-2 (BFI-2) [50]	Personality: open-mindedness, agreeableness, conscientiousness, extraversion, negative emotionality	60 items on a 5-point scale (1–5) from “disagree strongly” to “agree strongly” with 5 scales reported as the mean response. Open-mindedness scale evaluated as a primary measure and all others as secondary measures.
Visual Long-Term Potentiation Paradigm (EEG LTP) [55]	Plasticity: Hebbian plasticity	Participants are presented with visual stimuli which is “tetanised” with high-frequency stimulation, assessed as the amplitude of visual-evoked ERP response to tetanised stimuli vs non-tetanised stimuli.
Roving Mismatch Negativity Paradigm (EEG MMN) [56]	Plasticity: predictive coding	Participants are presented with a series of tones followed by a series of deviant tones, assessed as the amplitude of auditory-evoked ERP response to deviant tone and rate of subsequent habituation to tone.
Modified Tellegen Absorption Scale (MODTAS) [28, 51]	Personality: absorption	34 items rated on a 5-point scale (0–4) reported as the sum of scores (0–136).
Remote Associates Task (RAT) [52]	Creativity: convergent thinking	20 items; participants are given 3 words and need to produce a word that all 3 have in common, assessed as number correct and number attempted.
Resting-State fMRI	Connectivity	9-min continual recording on 3-T Siemens scanner with a 32-channel head coil.
Secondary measures		
5-Dimensional Altered States of Consciousness Questionnaire (5D-ASC) [57, 58]	Drug effects: psychological	91 items rated on VAS (1–100) with 5 scales and 11 subscales reported as % of the maximum score.
Adverse events	Unwanted health effects	Participants are asked daily to report any “unpleasant health issues” and to rate them as mild, moderate, or serious.
Consensual Assessment Technique (CAT) [59]	Creativity: non-specific	Participants are given 15 min to complete a paper collage; the result is rated by independent assessors on VAS (0–100) from low to high on (1) creativity and (2) technical goodness, reported as mean rater response.
Detail and Flexibility Questionnaire (DFlex) [60]	Attention to detail and cognitive rigidity	24 items on a 6-point scale (1–6) with 2 subscales reported as the sum of scores (total 24–144; subscales 12–72).
Daily questionnaire	Mood: well, sad, happy, stressed, creative, anxious, focused, tired, calm, connected, angry, energy, irritable, motivated, craving	15 items rated on VAS (0–100) reported as individual scores.
Depression, Anxiety, and Stress Scale (DASS) [61]	Mood: depression, anxiety, stress	42 items on a 4-point scale (0–3) with 3 subscales reported as the sum of scores (0–42).
Drug Effects Visual Analogue Scale (VAS)	Drug effects: psychological	16 items rated on VAS (0–100) reported as individual and mean scores.
Electrocardiogram (ECG)	Drug effects: physiological	QT interval, heart rate variability.
Everyday Problem-Solving Questionnaire	Creativity: problem solving	4 items rated on a VAS (0–100) reported as individual and mean scores.
Expectancy questionnaire	Expectancy	18 items rated on VAS (0–100; see Table 4).
Five Facets of Mindfulness Questionnaire (FFMQ) [62]	Mindfulness: observe, act with awareness, non-judgement, describe, non-reacting	39 items rated on a 5-point scale (1–5) with 5 subscales reported as the mean of scores.
Fitbit Charge 4, Activity and Sleep Tracker, manufactured by Fitbit, San Francisco, CA, USA	Drug effects: physiological	Activity reported as steps per day and sleep reported as minutes asleep per day.
Genetic biomarkers	Genetic	BDNF Val66Met.
Inflammatory cytokines	Immune modulation	Plasma concentration of inflammatory cytokines analysed reported in pg/ml.
NIH Toolbox Picture Vocabulary Test, Flanker Inhibitory Control and Attention Test, Picture Sequence Memory Test, List Sorting Working Memory Test, Dimensional Change Card Sort Test, Pattern Comparison Processing Speed Test [63]	Cognition: language, attention, executive function, episodic memory, working memory, processing speed	Reported as individual scores and NIH Fluid Cognition Composite Score Uncorrected Standard Score [64].
NIH Toolbox Anger-Affect, Anger-Hostility, Anger-Physical Aggression, Positive Affect, General Life Satisfaction, Meaning and Purpose, Emotional Support, Instrumental Support, Loneliness, Friendship, Perceived Hostility, Perceived Rejection, Self-Efficacy [65]	Mood: anger, positive affect, general life satisfaction, meaning and purpose, social support, companionship, social distress, self-efficacy	Reported as individual scores and NIH Psychological Well-being Summary, Social Satisfaction Summary [64].
Pharmacokinetic/pharmacodynamic (PKPD) measures	Drug metabolism	Plasma concentration of drug in pg/ml.
Peripheral mononuclear blood cell (PMBC) biomarkers	Physiology	5HT2A receptor mRNA expression in PMBC cells.
Profile of Mood States (POMS) [66]	Mood: fatigue, tension, depression, anger, vigour, confusion	65 items on a 5-point scale (0–4) with 6 subscales reported as the sum of scores.
Perceived Stress Scale (PSS) [67]	Mood: stress	10 items on a 5-point scale (0–5) reported as the sum of scores.
Self-Assessment Manikins (SAM) [68]	Drug effects: valence, arousal, dominance	3 items on a 9-point scale (1–9) reported as individual scores.
Semi-structured audio interview	Open-ended	~ 30-min interview with open-ended questions about the experience of microdosing trial for qualitative analysis.
Subject release interview	Open-ended	Brief discussion of participant’s experience of first dose and assessment of safety to discharge.
State of Surrender (SoS) [69]	Mindset: surrender	10 items on a 6-point scale (1–6) reported as the mean of scores.
State of Preoccupation (SoP) [69]	Mindset: preoccupation	4 items on a 6-point scale (1–6) reported as the mean of scores.
Vital signs	Physiology	Systolic and diastolic blood pressure in mmHg and heart rate in bpm.

### Participant recruitment

Advertisements will be placed on local noticeboards, on social media, and distributed via university mailing lists. Potential participants will be instructed to contact the study team via email. Participants will be compensated with $120 in supermarket vouchers for participating in the trial.

### Randomisation, masking, and code breaking

Eligible participants will be enrolled by a masked member of the study team. A biostatistician will perform the allocation of participants to either the active intervention or placebo (in blocks of ten at a 1:1 ratio) based on a computer-generated random sequence. Only the statistician and pharmacist members of the research team and the drug manufacturer will know the identity of the drugs to be administered (see the “Drug preparation and administration” section). Unmasked team members will not interact with study participants and will not be present during any drug administration sessions. The study team will be unmasked at the completion of each randomisation block; however, the participants will not be unmasked until the full completion of the trial.

### Drug preparation and administration

The study drug and placebo doses will be prepared by Biomed Ltd. (Auckland, New Zealand) to Good Manufacturing Practice (GMP) standards. The manufacturer will be supplied with randomisation codes for labelling each packet of five syringes by the study pharmacist. The study pharmacist will label and dispense the packets for each participant via a masked member of the study team. The study pharmacist will maintain an unmasked database of all participants. Emergency unmasking will be performed by the study pharmacist or their delegate in the case of a SAE or in any other scenario deemed necessary by the study clinicians.

### Strategies to improve adherence

Mobile Directly Observed Therapy (MDOT) will be used to monitor adherence to the home-dosing protocol. MDOT has been used recently in a variety of medication contexts including treatment of tuberculosis, HIV, opioid dependence, and asthma inhaler use [70]. Each dosing morning, participants will receive a text reminder with a link to upload a video of themselves self-administering. Participants will use their mobile phones to video themselves stating the date, displaying the sealed and labelled dose, then emptying the syringe into their mouth, holding their mouth closed for 30 s, and then providing a clear view of their empty mouth. Videos will be uploaded to a secure server to be checked by the study team and deleted immediately after viewing. Participants who fail to record the video correctly will be reinstructed, and those that fail to record repeatedly will be eliminated from the trial.

### Relevant concomitant care and post-trial care

Participants will receive care as normal from their GP during the trial and will be given guidelines of preferred treatments for any non-exclusionary health issues that occur within the trial. Long-term harm to participants is considered highly unlikely; however, participants will be able to apply for compensation for any injury sustained during the trial under the New Zealand Accident Compensation Corporation (ACC) scheme.

### Statistical analyses and power calculations

Given the novelty of the study, there are no effect sizes on which to base power calculations. The following power sensitivity calculations were performed in G*Power 3.1 [71] using *α* = 0.05, (1-*β*) = 0.8. For independent sample *t* tests where *n* = 80, our trial will be sensitive to the effects where Cohen’s *d* = 0.56. For linear regressions, *r =* 0.37 with *n* = 40 (single group) and *r* = 0.27 with *n* = 80 (combined groups). Dropouts are anticipated; however, due to the novelty of the protocol, the rate cannot be estimated. Dropouts and other instances of missing data will be accounted for in the analysis (see the “Sub-group data analysis and handling missing data” section). In the event that there are more than ten dropouts, an extra randomisation block of participants will be added to the study and the data added to per-protocol analyses.

Questionnaires and creativity tasks will be assessed with linear mixed models with participants modelled as random factors, using baseline measurements as a covariate. Analysis of language-based creativity tasks will additionally include fluency (as measured by the NIH Picture Vocabulary Test) as a covariate. EEG LTP and MMN data will be analysed consistent with Sumner et al. [53, 54]. fMRI data will be analysed for network connectivity, inter-network connectivity, and node-based connectivity, with an additional secondary analysis of seed-based functional connectivity of the left and right amygdala. Daily questionnaire VAS data will be analysed using a linear mixed effects model. Regression analyses of any significant effects will be undertaken with genetic biomarkers, baseline MODTAS scores, change in MODTAS scores, SoS/SoP, and fMRI baseline connectivity of the DMN as predictors. Expectancy effects will be assessed by regression analysis of measure scores to a corresponding construct on a baseline expectancy questionnaire (Table 4). Due to the use of multiple primary outcome measures, the Bonferroni-Holm step-down procedure will be employed to correct for multiple comparisons where appropriate. Secondary measures will be uncorrected but considered as exploratory.

**Table 4:** Expectancy items and corresponding measures

Expectancy item	Measures
*Do you expect that microdosing will change how ____ you feel?*	
Angry	Anger VAS
Anxious	Anxiety VAS DASS Anxiety (−) DFlex BFI-2 Negative Emotionality
Calm	Calm VAS
Connected to others	Connected VAS NIH Toolbox Social Satisfaction Summary BFI-2 Agreeableness BFI-2 Conscientiousness BFI-2 Extraversion
Creative	Creative VAS AUT RAT CAT Everyday problem solving
Focused	Focus VAS
Guilty	Guilt VAS
Happy	Happy VAS NIH Toolbox Psychological Well-Being Summary
Meditative	FFMQ
Motivated	Motivated VAS
Open to new experiences	BFI-2 Open-Mindedness MODTAS State of Surrender (−) State of Preoccupation 5D-ASC
Sad	Sad VAS DASS Depression
Stressed	Stressed VAS PSS DASS Stress
Well	Well VAS (−) AEs Fitbit Sleep and Activity
*Do you expect that microdosing will change how ____ you feel?*	
Craving	Craving VAS
Energy	Energy VAS (−) Tired VAS Fitbit Sleep and Activity
Self-efficacy	NIH Self-Efficacy
*Do you expect that microdosing will affect your ___*	
Cognitive functioning	NIH Toolbox Fluid Cognition Composite Score

“−” indicates negative correlation is expected

An interim analysis will be undertaken at 6-month periods for review by the Data Safety and Monitoring Committee (DSMC; see the “Data and safety monitoring” section). Interim analyses include recruitment and dropout rates, demographics, data completion, attendance and compliance, comparison of outcome measure baseline means by group, summaries of daily mood VAS ratings and vital signs, summaries of adverse events, and comparison of adverse events between the groups (see the “Adverse event reporting and harms” section). The DSMC is able to terminate the trial based on these reports.

### Sub-group data analysis and handling missing data

No pre-specified subgroup analyses are planned. Both per-protocol and intention-to-treat analyses with imputation will be calculated and checked for consistency in order to assess the impact of missing data.

### Adverse event reporting and harms

Participants will be prompted to report adverse events in their daily questionnaires and to rate these events as mild, moderate, or serious. These reports will be monitored daily by members of the study team. Participant rating of any adverse event as “serious” will trigger an immediate alert to the study team to follow up with the participant. Adverse events that occur during the study visits will be recorded by the members of the study team. Events judged to be a SAE by the study team will be reported to the DSMC within 5 working days of the event and to New Zealand Medicines and Medical Devices Safety Authority (Medsafe) as per their guidelines [72].

### Data and safety monitoring

The Trial Steering Committee (TSC) will provide overall supervision of the trial. The TSC will comprise all of the authors listed. In particular, the TSC will collaboratively develop and approve the final protocol; oversee the progress of the trial, adherence to the protocol, participant safety, and consideration of new information; and be responsible for publication and dissemination. The TSC must be in full agreement prior to submission of the final protocol. The TSC will take responsibility for the following, for which at least 50% of the investigators including the principal investigator (PI) must be in agreement: major decisions such as a need to change the protocol for any reason, monitoring and supervising the progress of the trial, and reviewing relevant information from other sources.

The study will be overseen by a DSMC provided by the Health Research Council of New Zealand (HRC), the primary funders of this study. The DMSC consists of two biostatisticians, several clinicians, and an ethicist. Reports will be submitted to the DMSC every 6 months for review of trial progress and conduct. Protocol amendments will be submitted to the DSMC, as well as Medsafe and the approving Ethics Committee.

### Data collection and management

Separate paper-based files will be kept for each participant, while the bulk of the case report form (CRF) and data capture will be managed with the online Research Electronic Data Capture (REDCap) tools hosted at the University of Auckland [73]. REDCap is a secure, web-based software platform designed to support data capture for clinical trials. Demographics, medical history, height, weight, MRI screening, current medications, notes on physical examinations, vital signs, drug/alcohol screening results, daily questionnaires, adverse events recorded at the study site, eligibility confirmation, and all self-reported questionnaires will be entered directly into REDCap. The psychological screening assessments will be completed on paper and appended to the paper CRF. Electrocardiogram (ECG) results will be printed and appended to the paper CRF. EEG, MRI, NIH Cognitive and Emotional Batteries, and Sleep and Activity tracking data will all be captured electronically. Serum chemistry and haematology, biomarker, and pharmacokinetic data will all be received in electronic format from subcontracted laboratories. In the case of a Covid-19 lockdown occurring during the trial, all data which can be captured remotely will still be collected, including all questionnaires, NIH Toolbox assessments, creativity tasks, and interviews.

All electronic data will be identified only by participant number and stored on secure University of Auckland servers which include password protection, multi-site backups, and tape archiving. An original, unprocessed version of every data file will be kept on the servers such that these files may only be modified by a University of Auckland IT systems administrator—thus ensuring the fidelity and audit capability of all electronic data. Scanned versions of all paper-based CRFs and source data formats will be made and held on the servers in password-protected files to ensure fidelity of these data and allowing future audit of extracted data. Biological specimens will be stored for analysis at the University of Auckland and will not be used for any future studies. As outlined in the PIS, biological samples or identifiable medical data will be shared with any party outside of the study team; however, deidentified data may be shared with other researchers.

### Dissemination policy

Results will be published in relevant academic journals and will be communicated with the wider public via news media and social media. Participants will be able to view their own data.

## Discussion

This study will provide one of the first opportunities to assess the effects of long-term psychedelic microdosing in a naturalistic environment using objective measures, placebo controls, standardised doses of a psychedelic, and with a thorough examination of expectancy and unmasking during the trial. A comprehensive battery of objective and subjective validated measures, as well as qualitative interviews, will give a wide view of the effects of microdosing across a breadth of cognitive and psychological domains. Collecting EEG measures at the acute phase of a single dose and at the end of a 6-week course of regular microdosing will indicate whether plasticity changes potentially mediate the reported effects of microdosing and whether these changes persist outside of the drug’s presence in the body and accumulate over time. Analysis of resting-state networks measured by fMRI will allow us to see whether similar functional alterations to those seen under much larger doses of psychedelics are present following microdoses and enhance understanding of psychedelic effects overall.

Careful monitoring of expectancy and unmasking will also give insight into the role of placebo effects in full-dose psychedelic experiences. Due to the significant perceptual effects of psychedelics, placebo effects are difficult to parse in full dose trials; however, sub-hallucinogenic microdoses that are carefully monitored for unmasking will provide an opportunity to gain an insight into the magnitude of these effects. Expectancy of the positive effects of microdosing has been demonstrated among those who frequent drug use forums [20], likely inflating the benefits reported by those who learn about microdosing online, and these attitudes have been shown to correlate to positive self-reported outcomes [13], and to potentially explain the difference in outcomes between microdosing and placebo in a self-blinded study of people microdosing in the community [21]. Positive coverage of the practice in local news will potentially be producing a similar effect in the general population from which we are drawing participants. By assessing expectancy at baseline, we will be able to assess the effect of these attitudes on participant outcomes. Expectancy is also likely to change in uncontrollable ways during the trial, as participants may be exposed to information about microdosing and talk to peers about the trial. Efforts need to be made to reduce these occurrences, for example, by keeping participants with concurrent appointments separate from each other and by discouraging participants from conducting online research into microdosing. Efforts will also be made to prevent unmasking and nocebo effects by priming participants with the expectation that they may not feel any immediate effect from the dose. Participants will not be unmasked until the conclusion of the trial, so that they cannot share whether their experiences were due to placebo or an active dose with associates who may also be enrolled in the trial.

Beyond testing the efficacy and safety of the practice on healthy participants, the practical implementation of a home-dosing regimen of a restricted and easily degraded drug is an essential aspect of assessing the feasibility of LSD microdosing as a potential mental health treatment. Analysis of participant adherence and compliance using MDOT will be valuable to the planning of future trials on clinical populations, as well as shaping policy around potential access in clinical settings.

### Trial status

The MDLSD protocol is currently on version 3.0. Recruiting of this trial is due to commence on April 12, 2021, and run through to the anticipated completion of the trial in mid-2022.

## Supplementary Information

**Additional file 1.** Copy of the participant information sheet and informed consent form.

## Data Availability

The corresponding author will release documentation including full protocol, PIS, consent forms, and study advertisements on publication of trial results. Access to the final trial dataset will only be available to the study investigators, DSMC, and any other relevant regulatory bodies. Statistical code and de-identified datasets will be made available upon reasonable request.

## Abbreviations

5D-ASC: Five Dimensions of Altered States of Consciousness Questionnaire
5HT: 5-Hydroxytryptamine (serotonin)
ACC: New Zealand Accident Compensation Corporation
ADHD: Attention-deficit hyperactivity disorder
AE: Adverse events
ANCOVA: Analysis of covariance
AUT: Alternate Uses Test
BDNF: Brain-derived neurotrophic factor
BFI-2: Big Five Inventory-2
C-SSRS: Columbia-Suicide Severity Rating Scale
CAARMS: Comprehensive Assessment of At Risk Mental States
CAT: Consensual assessment technique
CRF: Case report form
DASS: Depression Anxiety Stress Scales
DFlex: Detail and Flexibility Questionnaire
DMN: Default Mode Network
DOI: 2,5-Dimethoxy-4-iodoamphetamine
DSMC: Data and Safety Monitoring Committee
EEG: Electroencephalography
FFMQ: Five Facets of Mindfulness Questionnaire
fMRI: Functional Magnetic Resonance Imaging
GMP: Good Manufacturing Practice
GP: General practitioner
HRC: Health Research Council of New Zealand
LSD: Lysergic acid diethylamide
LTP: Long-term potentiation
MDOT: Mobile direct observation of therapy
Medsafe: New Zealand Medicines and Medical Devices Safety Authority
MINI: Standard Mini-International Neuropsychiatric Interview
MMN: Roving mismatched negativity
MODTAS: Modified Tellegen Absorption Scale
NIH: National Institute of Health
NM-ASSIST: NIDA-Modified Alcohol, Smoking, and Substance Involvement Screening Test
PI: Principal investigator
PIS: Participant information sheet
PKPD: Pharmacokinetic/pharmacodynamic
PMBC: Peripheral mononuclear blood cells
PTSD: Post-traumatic stress disorder
PSS: Perceived Stress Scale
RAT: Remote Associates Task
SAE: Serious adverse event
SAM: Self-Assessment Mannikins
SoS/SoP: State of Surrender/State of Preoccupation
TAS: Tellegen Absorption Scale
TSC: Trial Steering Committee
VAS: Visual analogue scale
