# Correction to: MDLSD: study protocol for a randomised, double-masked, placebo-controlled trial of repeated microdoses of LSD in healthy volunteers

**DOI:** 10.1186/s13063-021-05307-4

**Published:** 2021-05-10

**Authors:** Robin J. Murphy, Rachael L. Sumner, William Evans, David Menkes, Ingo Lambrecht, Rhys Ponton, Frederick Sundram, Nicholas Hoeh, Sanya Ram, Lisa Reynolds, Suresh Muthukumaraswamy

**Affiliations:** 1 School of Pharmacy, Faculty of Medical and Health Sciences, University of Auckland, 85 Park Road, Grafton, Auckland, 1023 New Zealand; 2 Mana Health, 7 Ruskin St, Parnell, Auckland, 1052 New Zealand; 3 Department of Psychological Medicine, Faculty of Medical and Health Sciences, Waikato Clinical Campus, Peter Rothwell Academic Centre, University of Auckland, Pembroke Street, Hamilton, 3240 New Zealand; 4 Regional Cancer & Blood Service, Auckland District Health Board, 2 Park Road, Grafton, Auckland, 1023 New Zealand; 5 Department of Psychological Medicine, Faculty of Medical and Health Sciences, University of Auckland, 2 Park Road, Grafton, Auckland, 1023 New Zealand; 6 Department of Psychological Medicine, Faculty of Medical and Health Sciences, University of Auckland, 22-30 Park Avenue, Grafton, Auckland, 1023 New Zealand

**Correction to: Trials 22, 302 (2021)**

**https://doi.org/10.1186/s13063-021-05243-3**

Following the publication of the original article [1], we were notified that the title of Table 2 should be ‘Table 2 Full exclusion criteria’.

The original article has been corrected.
