# Supplementary material for: MDLSD: study protocol for a randomised, double-masked, placebo-controlled trial of repeated microdoses of LSD in healthy volunteers
Source: Trials. 2021 Apr 23;22:302. doi: 10.1186/s13063-021-05243-3 (PMC8062934; doi:10.1186/s13063-021-05243-3)
Supplement: Supplementary file 1 — Additional file 1. Copy of the participant information sheet and informed consent form. [file 13063_2021_5243_MOESM1_ESM.pdf]

THE UNIVERSITY OF AUCKLAND
Te Whare Wananga o Tamaki Makaurau
NEW ZEALAND

Dr Suresh Muthukumaraswamy
Associate Professor
The University of Auckland
Private Bag 92019
Auckland
New Zealand

Building 505, Level 3, 85 Park Road, Grafton
Auckland 1142, New Zealand

Telephone: 64 9 373 7599 Ext 85398
Facsimile: 64 9 367 7192
Email: sd.muthu@auckland.ac.nz

Participant Information Sheet

Study title:	**Microdosing LSD**
Locality:	**The University of Auckland**
Lead investigator:	**Dr Suresh Muthukumaraswamy**
Ethics committee ref.:	**19/STH/91**
Contact phone number:	**(09) 373 7599 x85398**

You are invited to take part in a study on the effects of repeated microdoses of LSD on the brain and behaviour. Whether or not you take part is your choice. If you don't want to take part, you don't have to give a reason, and it won't affect any future studies you may wish to do with us. If you do want to take part now, but change your mind later, you can pull out of the study at any time.

This Participant Information Sheet will help you decide if you'd like to take part. It sets out why we are doing the study, what your participation would involve, what the benefits and risks to you might be, and what would happen after the study ends. We will go through this information with you and answer any questions you may have. You do not have to decide today whether or not you will participate in this study. Before you decide you may want to talk about the study with other people, such as family, whānau, friends, or healthcare providers. Feel free to do this.

If you agree to take part in this study, you will be asked to sign the Consent Form on the last page of this document. You will be given a copy of both the Participant Information Sheet and the Consent Form to keep.

This document is 10 pages long, including the Consent Form. Please make sure you have read and understood all the pages.

WHAT IS THE PURPOSE OF THE STUDY?

- In this study we are investigating whether very small doses of LSD have effects on the brain and behaviour. In large doses LSD has psychedelic properties which include temporary alterations or distortions in perception, heightened awareness of surroundings, and profound spiritual experiences. Taking very small doses of LSD is

called microdosing and does not have psychedelic effects. However, many users claim that taking regular microdoses of LSD can have positive effects on personality, creativity and mood. However, this has not been tested in a formal study and these claimed effects may or may not be real. It has also been claimed that microdosing with LSD might be beneficial for people suffering from mood disorders such as depression or anxiety.

- The goal of our research is to test whether taking microdoses of LSD does have measurable effects on brain and behaviour in healthy individuals as claimed by users. As such, your participation will help us test these claims.
- This study is a placebo-controlled trial, meaning that some of the doses administered will be LSD and some will be inactive placebo.
- We are a group of scientists and clinicians based at the University of Auckland and the Auckland District Health Board who are studying the effects of drugs on the brain. This study is being done as part of a student's PhD thesis. This study has been approved by a Health and Disability Ethics Committee. Contact details are given at the end of this sheet.
- This study is funded by a philanthropic donations and the Health Research Council of New Zealand.

WHAT WILL MY PARTICIPATION IN THE STUDY INVOLVE?

- You are reading this sheet because you have seen advertisements and you have contacted us.
- This study will take up to 20 hours of your time and involve four visits to our research centre. We describe these visits below and have added a table at the bottom of this section to help you understand the timing of the visits and what happens at each visit.
- On the first day you will come to our centre and we will confirm your eligibility to take part in the study. This session will take approximately two and a half hours. We will ask you questions about your physical and mental health as well as drug use history to confirm whether you are eligible to take part. We will take urine samples to test you for recent use of illicit drugs and blood samples to test your physical health. A physical health assessment will be performed including an ECG recording of your heart.
- Most visits will involve you giving us blood and urine samples for analysis. You need to be comfortable with needles and giving blood.
- In order to take part in the study you must consent for us to use genetic information contained in the samples you provide us. Our genetic analysis of the blood samples that you provide us will be used to see if particular genes related to brain and liver function affect the response to the drugs that we give you. Our use of the genetic information you provide us will be strictly limited for the purposes of the current study and will not be used for any diagnostic and/or ancestry testing. Please ask us any questions you may have about this.
- Any samples that you provide us will not leave New Zealand.
- If you are Māori, you are encouraged to consult with your whanau, hapu or iwi regarding participation in this project. You may hold beliefs about a sacred and shared value of all or any tissue samples removed. The cultural issues associated with sending or storing your tissue should be discussed with your family/whanau as appropriate. There are a range of views held by Māori around these issues; some iwi disagree with storage of samples citing whakapapa and advise their people to consult

prior to participation in research where this occurs. However, it is acknowledged that individuals have the right to choose.

- If you are eligible and decide to take part we will schedule the upcoming three study visits and two resupply days.
- We will also provide you a safety card with information on whom to contact should you have any health concerns during the study. We will make sure these details are on your mobile phone also.
- On your second visit we will conduct further tests on you. When you come in for the second visit we will collect information about your brain activity using our brain scanners (MRI and EEG). More details about EEG and MRI are given below. We will also ask you to complete a number of psychological questionnaires and perform some simple tasks. This day will take approximately four hours.
- At the end of this visit we will give you a FitBit to wear for the rest of the study (day and night) so that we can collect data on your sleeping and activity patterns throughout the study. The FitBit App will be installed on your phone and you will need to charge the FitBit approximately every 7 days The GPS on your FitBit will be turned off to maintain your privacy and no personally identifiable information about you will be entered into the FitBit app. Nevertheless, you should be aware that your de-identified data will be held by FitBit and subject to their data policies (https://www.fitbit.com/nz/legal/privacy-policy). FitBit may process your data, however, we will delete your data off the FitBit website when the study is finished. You will need to return the FitBit to us at the end of the study. Please ask us if you are unsure about this part of the study.
- When you go home you will also be texted a link to a quick questionnaire to complete every night on your phone. This questionnaire asks you about your daily experiences, mood and health status. We ask that you complete the questionnaire by 9pm. If you forget to complete your nightly questionnaire we will text and, if necessary, phone to remind you. Because of this, you need to have a working smart phone with data which you check often in order to participate in this trial. If at any point you cannot afford to top up your phone data, contact the study team and we can reimburse you for this cost.
- On your third visit you will receive your first microdose of either LSD or a placebo. The dose is taken as an oral syringe (this does not involve a needle, the dose is squirted underneath your tongue). This will be done under the clinical supervision of a doctor or nurse, and your health, heart rate and blood pressure will be checked for six hours before can go home. We ask that you do not drive yourself to the clinic on this day and instead arrange to be picked up by a friend, or else we will arrange a taxi for you. Six blood samples will be taken from you during this time-period (10 ml each – two teaspoons) so we can later measure how much of the drug is in your blood. In this study the placebo will be an inactive oral syringe.
- When you go home you will be given the drug to take yourself for the next three weeks. You will take the drug every 3rd morning. We will send you text messages to remind you. The oral syringe box you will be given with the medicines will have clear instructions on it. We will ask you to upload a video of yourself taking the dose. This video will go directly onto a secure server, will only be viewed by the study team, and will be deleted as soon as it is confirmed that you have taken the dose. It is especially important that you complete the nightly questionnaires on the dose days.

- After two and four weeks you will need to revisit us to receive more of the drug. At these visits we will give you a brief health check. These visits will take approximately 10 minutes.
- After the six-week period is over you will then visit us for a fourth time and we will repeat the EEG/MRI/ECG and psychological tests that you completed on your second visit. This visit will take approximately four hours.
- After one month and three months we will ring you for a phone follow-up to check on your health. This call will take only ten minutes.
- At the end of the study (which may be up to 18 months from when you started) we will ring you to debrief and tell you which doses were LSD/placebo.

**Study Timeline**	**What will happen?**	**Duration**	**When**
Visit 1 - Screening	Medical history<br>Questionnaires<br>Medical exam/questions<br>ECG<br>Blood samples<br>Urine testing	2.5 hours	Week 1
Visit 2 - Measures	Medical exam/questions<br>Questionnaires<br>EEG<br>MRI<br>Urine testing	4 hours	Week 1
Visit 3 - Microdosing	Medical exam/questions<br>Questionnaires<br>Drug dose (any dose could be LSD or placebo)<br>EEG<br>Blood samples<br>Urine testing,	8 hours	Week 2
	Dose at home<br>(any dose could be LSD or placebo)		Weeks 2- 7
Resupply 1	Brief health check	10 minutes	Week 3-4
Resupply 2	Brief health check	10 minutes	Week 5
Visit 4 - Measures	Medical exam/questions<br>Questionnaires<br>EEG<br>MRI<br>Urine testing<br>Blood samples	4 hours	Beginning Week 8
	Follow up	10 minutes	1/3 months

WHAT DOES BRAIN SCANNING INVOLVE

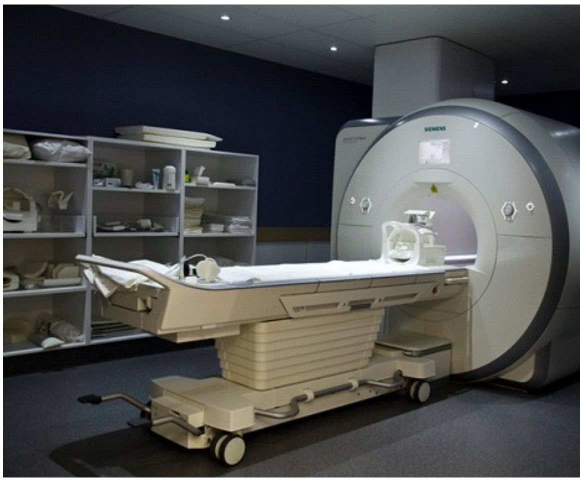

The Auckland University MRI scanner

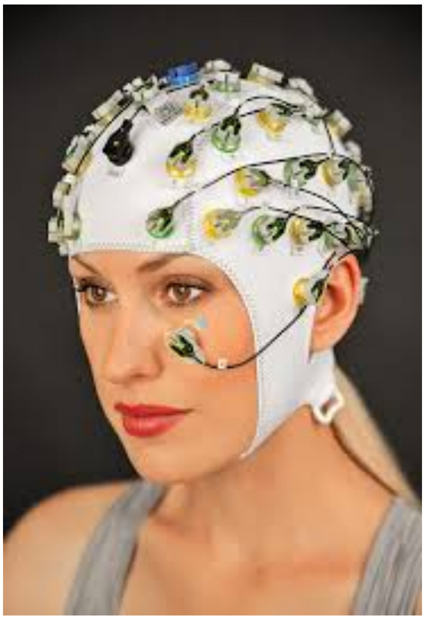
The Auckland University EEG cap

- For the MRI scans you will change into clothes we will provide for you. We will check that you have no metal on your body before you enter.
- The MRI scanner is very loud and for some people can feel very enclosed. As such, it can be a little scary if you have not been in one before. We will give you headphones to protect your hearing. Let us know if you feel uncomfortable or apprehensive in any way. You will be given an emergency buzzer that you can press so that you can leave the scanner at any time during the procedure
- The MRI scan will take up to thirty minutes. We will ask you to lie as still as possible during the scans
- For the EEG scan you can wear your normal clothes. To set up the EEG scan we will place a cap of electrodes over your head. This will take around 30 minutes to set up. To make the set-up easier, please come to the study days with clean and dry hair with no hair-care products in your hair.
- The EEG scan will take up to an hour. There will be some simple tasks for you do.

WHAT ARE THE POSSIBLE BENEFITS AND RISKS OF THIS STUDY?

- There may be no direct benefits to you in taking part in this study.
- In the event that a condition which is assessed to be a clinical abnormality is detected through performing a scan on you, you will be informed of this - should you consent. Your general practitioner or other health professional of your choice must be notified. The images are not routinely reviewed by a radiologist, therefore we are unable to perform diagnostic scans for medical purposes. As such, you should not assume that if there is no brain abnormality reported to you and/or your chosen health professional, that you have no brain abnormality.
- You should be aware that once you have been informed that a clinical abnormality has been detected through performing a scan on you this could affect your ability to obtain insurance whether or not you take the matter further.
- The drug that we will use in this study are given at very low doses. We will be careful and your first dose will be given under clinical supervision. The LSD you will be given

will be prepared by a pharmaceutical company under strict manufacturing conditions to ensure the drug is pure.

- A previous study using this microdose of LSD found some participants reported mild/moderate intensity headaches. You might also experience these. If you do let us know so we can advise you.
- Blood samples will be obtained through an intravenous (IV) cannula. An IV cannula is a small flexible tube put into a vein in your arm, the same as if you have been on a drip in a hospital. This can cause side-effects. The most common of these are bruising, mild pain and feeling faint when it is put in.
- Although the doses of the drug given are very low and should not have any perceptible effects **we advise that you do not drive, operate machinery, or engage in any dangerous activities or important care giving roles where you could put yourself or others in harm's way for six hours after taking a dose.** Do not change these plans, even if you do not feel any effects or have not felt any effects so far. Any dose that you receive could contain LSD or a placebo and you may not be able to feel any obvious effects, so it is essential that you maintain these arrangements for every dose day.
- **You should be aware that LSD is a Class A controlled substance**. Although given at very low doses **taking this regularly may be incompatible with your employment conditions**. You should discuss this with your employer and/or the study team before starting the study.
- **You should keep the study drugs safe, out of the reach of children and should not share them with others. You should take them exactly as requested. It may be unsafe for you to not do so. Please discuss this with the study team.**
- You should be aware that the LSD being given to you will not be available to you after the study is finished. LSD is an illegal Class A drug in New Zealand.

WHO PAYS FOR THE STUDY?

We will reimburse you for any reasonable travel and food costs relating to the study, as well as phone credit if necessary. We will also buy any food you need while you are taking part in the study and visiting us. If you require a taxi to get to and from the study then we can arrange and pay for this. Please keep any receipts so that we can give you vouchers for your costs. We recognise that taking part in the study will take up to twenty hours of your time and will provide you with $120 of vouchers at the end of the study in recognition of this inconvenience.

WHAT IF SOMETHING GOES WRONG?

If you were injured in this study, you would be eligible **to apply** for compensation from ACC just as you would be if you were injured in an accident at work or at home. This does not mean that your claim will automatically be accepted. You will have to lodge a claim with ACC, which may take some time to assess. If your claim is accepted, you will receive funding to assist in your recovery.

If you have private health or life insurance, you may wish to check with your insurer that taking part in this study won't affect your cover.

WHAT ARE MY RIGHTS?

- Your participation in this study is entirely voluntary. It is up to you if you take part or not. If you do decide to take part you will be given this information sheet to keep and be asked to sign a consent form. If you don't want to take part you don't have to give a reason. If you decide to take part you are still free to withdraw at any time and without giving a reason. A decision to withdraw at any time, or a decision not to take part, will not affect the standard of care you receive from us. However, if you do withdraw from the study prematurely, for your benefit we will ask you to remain at our research facility until it is safe for you to leave.
- If you take part in the study you have the right to access any information about you collected during the study after your completion or withdrawal from the study.
- If we learn anything about your health status during the study that affects your health you will be informed of this.
- Any information you give us about yourself will remain private and confidential and only can be seen by members of the research team.

WHAT HAPPENS AFTER THE STUDY OR IF I CHANGE MY MIND?

- At the conclusion of the study your personal data will be kept in locked cabinets in secure rooms at the University of Auckland and kept for ten years. They will be shredded after this time. Your data will be identified by a unique trial specific number in any database. Your name will not be included in any trial data electronic file.
- Most electronic data files will be kept in a de-identified format such that there is no risk that you could be identified from these data. Some data such as interviews and voice recordings are potentially identifiable from the electronic files. These data will be kept in password protected files.
- Some of your data may be sent to overseas collaborators who will sign confidentiality agreements with us to use the data. Only members of the study team and appropriate regulatory bodies will be able to access your blood samples and identifiable health information.
- Any blood samples that you give us will be destroyed after they have been analysed.
- It can take quite a long time for us to analyse data from these kinds of studies. We hope to be able to tell you the final results one to two years after completion of the study. We plan to publish the results in specialised academic journals. If you want us to we can send you a summary of the results in an easier format to read.
- At any stage, during or after the study you are entitled to a complete copy of your data should you wish. In order to for us to do this you will need to let us know and visit us separately and provide us with an empty 8 GB USB flash drive. This is necessary because your data set will be too large to email and we need to ensure it is securely transferred.

WHO DO I CONTACT FOR MORE INFORMATION OR IF I HAVE CONCERNS?

If you have any questions, concerns or complaints about the study at any stage, you can contact:

Dr Suresh Muthukumaraswamy, Associate Professor
Phone: 09 373 7599 ext 85398

Email: sd.muthu@auckland.ac.nz

If you want to talk to someone who isn't involved with the study, you can contact an independent health and disability advocate on:

Phone:	0800 555 050
Fax:	0800 2 SUPPORT (0800 2787 7678)
Email:	advocacy@hdc.org.nz

For Maori health support please contact :

He Kamaka Waiora (Maori Health Team)
Telephone number: 09 486 8324 x 2324
Email: hkw@adhb.govt.nz

You can also contact the health and disability ethics committee (HDEC) that approved this study on:

Phone:	0800 4 ETHICS
Email:	hdecs@moh.govt.nz

THE UNIVERSITY OF AUCKLAND
Te Whare Wananga o Tamaki Makaurau
NEW ZEALAND

Dr Suresh Muthukumaraswamy
Senior Lecturer
The University of Auckland
Private Bag 92019
Auckland
New Zealand

Building 505, Level 3, 85 Park Road, Grafton
Auckland 1142, New Zealand

Telephone: 64 9 373 7599 Ext 85398
Facsimile: 64 9 367 7192
Email: sd.muthu@auckland.ac.nz

Consent Form

Please tick to indicate you consent to the following

I have read, or have had read to me in my first language, and I understand the Participant Information Sheet.	Yes ☐
I have been given sufficient time to consider whether or not to participate in this study.	Yes ☐
I have had the opportunity to use a legal representative to discuss my participation in this study	Yes ☐
I have had the opportunity to use a whanau/ family support or a friend to help me ask questions and understand the study.	Yes ☐
I am satisfied with the answers I have been given regarding the study and I have a copy of this consent form and information sheet.	Yes ☐
I understand that taking part in this study is voluntary (my choice) and that I may withdraw from the study at any time without this affecting my medical care from the study team.	Yes ☐
I consent to the research staff collecting and processing my information, including information about my health.	Yes ☐
If I decide to withdraw from the study, I agree that the information collected about me up to the point when I withdraw may continue to be processed.	Yes ☐
I consent to my GP or current provider being informed about my participation in the study and of any significant abnormal results obtained during the study.	Yes ☐
I agree to my blood samples will be disposed of using established guidelines for discarding biohazard waste.	Yes ☐
I agree to an approved auditor appointed by the New Zealand Health and Disability Ethic Committees, or any relevant regulatory authority or their approved representative reviewing my relevant medical records for the sole purpose of checking the accuracy of the information recorded for the study.	Yes ☐
I understand that my participation in this study is confidential and that no material, which could identify me personally, will be used in any reports on this study.	Yes ☐

I understand the compensation provisions in case of injury during the study.	Yes ☐	
I know who to contact if I have any questions about the study in general.	Yes ☐	
I understand my responsibilities as a study participant.	Yes ☐	
**I will not take part in dangerous activities for six hours after taking drug doses.**	Yes ☐	
**I will take the drugs as directed, will keep them out of reach of children and will not provide them to any other persons.**	Yes ☐	
I understand that LSD is a Class A controlled substance remains the property of the University of Auckland and must be returned if requested and should only be used as directed. I understand that failure to do these things may be a criminal offence and the New Zealand police may be contacted.	Yes ☐	
I wish to receive a summary of the results from the study.<br><br>If yes provide contact details (email)	Yes ☐	No ☐

Declaration by participant:
I hereby consent to take part in this study.

Participant's name:

Signature: Date:

Declaration by member of research team:

I have given a verbal explanation of the research project to the participant, and have answered the participant's questions about it.

I believe that the participant understands the study and has given informed consent to participate.

Researcher's name:

Signature: Date:
